# KangPiLao decoction modulates cognitive and emotional disorders in rats with central fatigue through the GABA/Glu pathway

**DOI:** 10.3389/fphar.2022.939169

**Published:** 2022-09-02

**Authors:** Yifei Xu, Yajun Lian, Jie Li, Yifei Zhang, Yan Liu, Xuejiao Wang, Jie Ma, Feng Li

**Affiliations:** ^1^ Beijing University of Chinese Medicine, Beijing, China; ^2^ Nanjing University of Chinese Medicine, Nanjing, China; ^3^ Guang’anmen Hospital, China Academy of Chinese Medical Sciences, Beijing, China

**Keywords:** chinese herbal medicine, γ-aminobutyric acid, glutamic acid, central fatigue, chronic fatigue, cognitive disorder, mood disorders

## Abstract

**Background:** Central fatigue (CF) is a subjective sense of tiredness associated with cognitive and memory disorders, accompanied by reduced physical endurance and negative emotions, such as anxiety and depression. Disease progression and prognosis with regards to CF have been unfavorable and possibly contribute to dementia, schizophrenia, and other diseases. Additionally, effective treatments for CF are lacking. KangPiLao decoction (KPLD) has been widely applied in clinical treatment and is composed of six Chinese herbal medicines, some of which have confirmed anti-fatigue effects. While glutamic acid (Glu) is the main excitatory transmitter in the central nervous system (CNS), gamma-aminobutyric acid (GABA) is the major inhibitory transmitter. Both are involved in emotional, cognitive, and memory functions. This research was designed to explore how KPLD regulates cognitive and emotional disorders in rats with CF and to identify the relationship between the regulatory effect and the GABA/Glu pathway.

**Methods:** The compounds comprising KPLD were analyzed using high-performance liquid chromatography-mass spectrometry. Sixty Wistar rats were randomly divided into six groups. The modified multiple platform method was used to induce CF. Cognitive, emotional, and fatigue states were evaluated by performing behavioral tests (Morris water maze [MWM], open-field test [OFT], and grip strength test). Histomorphology, western blotting, immunohistochemistry, and RT-qPCR were performed to investigate protein and mRNA expression levels in the hippocampus and prefrontal cortexes involved in the GABA/Glu pathway.

**Results:** Rats with CF exhibited impaired spatial cognition and increased negative emotions in the MWM and OFT. KPLD enabled the improvement of these symptoms, especially in the high-concentration group. Western blotting and RT-qPCR demonstrated that the expression of GABAARα1, GABAARγ2, GABABR1, and GAD67 in rats with CF was higher, whereas GAT-1 and NMDAR2B were lower in the hippocampus and prefrontal cortex. KPLD decreased the expression of GABAARα1, GABABR1, GABAARγ2, and GAD67 in the hippocampus and prefrontal cortex and enhanced the expression of NR2B in the prefrontal cortex.

**Conclusion:** KPLD significantly improved cognitive and emotional disorders in rats with CF by regulating the GABA/Glu pathway. Overall, KPLD may be a promising candidate for developing a drug for treating CF.

## 1 Introduction

Fatigue is a symptom or comorbidity of a neurological disorder. Studies have found that more than 50% of people feel fatigued, and more than one-third of them clearly believe that they are affected by fatigue, which seriously reduces their quality of life and productivity (Ishii et al., 2014; Penner and Paul, 2017). As early as 1904, Italian physiologist, A. Mosso, discovered that fatigue could occur after thinking tasks such as lectures and consequently introduced the concept of CF (Dalsgaard and Secher, 2007). Unlike peripheral fatigue (Nybo and Secher, 2004), The Lancet proposes that CF is not just a feeling of physical exhaustion but a cognitive impairment with limited ability to maintain focus and withstand mental tasks (Chaudhuri and Behan, 2004; Ayache and Chalah, 2021). Cognitive fatigue may manifest as 1) subjective fatigue sensations or negative emotional feelings and 2) a decline in learning, cognition, and memory performance (Lindqvist and Malmgren, 1993; Kluger et al., 2013). CF can aggravate the disease course over time, and the cognitive impairment associated with the early stage of CF could progress to dementia (Rajan et al., 2015; Sander et al., 2016). As such, early intervention of CF is of great significance.

Studies have shown that CF may be related to abnormal regulation of the immune system (Nakatomi et al., 2014) or neurotransmitter systems (Davis et al., 2000; Newsholme and Blomstrand, 2006), nerve pathway conduction damage (Leavitt and Deluca, 2010), and energy expenditure (Jason et al., 2010). However, the mechanism underlying CF remains unclear. Glutamic acid (Glu) and gamma-aminobutyric acid (GABA) are the brain’s principal excitatory and inhibitory neurotransmitters, respectively. They are associated with cognitive functions, such as memory, learning, and emotional control (Akerman and Cline, 2007; Tessier and Broadie, 2009). Glu is essential for the functional transmission and processing of information within the CNS (Lynch, 2004). Astrocytes are the main site for maintaining the level of GABA transporters and are inseparable from the formation of memory. GABA is associated with fatigue (Riekki et al., 2008), and the neuronal activity related to learning and cognitive function is modulated by GABAergic neurons, with significant effects on information processing, plasticity, and network synchronization (Bhat et al., 2010).

The hippocampus and prefrontal cortex may be the critical sites involved in cognitive and emotional changes in CF. Studies have confirmed that the hippocampus is closely related to emotional cognitive activity (Tannenholz et al., 2014) and that the prefrontal cortex processes higher cognitive functions (Arnsten, 2015). Sepulcre et al. (2009) found that CF in multiple sclerosis was mainly distributed in the frontal and temporal lobes, as well as other brain regions. Functional magnetic resonance imaging scans also provided evidence that functional brain activation of the frontal lobe was markedly reduced in fatigued patients (Filippi et al., 2002). However, relevant studies focusing on the cognitive and emotional disorders of CF are limited. Therefore, the changes in the GABA/Glu mechanism in the hippocampus and prefrontal cortex in CF require further investigation.

There are no specific drugs for the treatment of CF. The currently reported treatment regimens mainly include: 1) energy supplement preparations, e.g., CoQ10 (Hargreaves, 2014; Maguire et al., 2020), L-carnitine (Malaguarnera et al., 2007), creatine monohydrate (Watanabe et al., 2002), etc.; 2) Neurotransmitter-modulating drugs: dopamine and norepinephrine analogs, such as amphetamine (Sacks et al., 2018), serotonin reuptake inhibitors and excitatory transmitters (Meeusen et al., 2006), which require long-term use and result in severe drug dependence and withdrawal symptoms (Meng et al., 2020); 3) Complementary alternative therapy, such as aromatherapy (Sanoobar et al., 2016), hot baths, yoga, and music therapy (Varney and Buckle, 2013); or 4) Nutritional supplements such as branched-chain amino acids, carbohydrates, etc. A systematic review found that carbohydrates attenuated CF; however, direct evidence for this is limited (Khong et al., 2017).

Overall, effective treatments for CF are lacking. Studies have confirmed that traditional Chinese medicine can significantly improve fatigue (Tharakan and Manyam, 2006), such as *Panax ginseng* C.A.Mey [Araliaceae] (Bao et al., 2016), *Astragalus mongholicus* Bunge [Fabaceae] (Liu et al., 2017), and *Angelica sinensis* (Oliv.) Diels [Apiaceae] (Yeh et al., 2014). Based on safety and the potential application scope of the drug, we selected medicine and food homologous herbal varieties that are known to contain anti-fatigue ingredients in order to prepare the KangPiLao decoction (KPLD), which has been shown to possess fatigue attenuation effects (Li et al., 2015; Han et al., 2018a). KPLD has been widely used in the clinical treatment of fatigue and is composed of six Chinese herbal medicines, including *Astragalus mongholicus* Bunge [Fabaceae], *Angelica sinensis* (Oliv.) Diels [Apiaceae], *Dendrobium officinale* Kimura & Migo [Orchidaceae], *Citrus* × *aurantium* L. [Rutaceae], *Crataegus pinnatifida* Bunge [Rosaceae], and *Schisandra chinensis* (Turcz.) Baill. [Schisandraceae]. The aim of this study was to investigate the effects of KPLD on cognitive and emotional disorders and whether these effects are regulated by the GABA/Glu pathway in the brain.

## 2 Materials and methods

### 2.1 Preparation and analysis of KangPiLao decoction

The ingredients of KPLD were purchased from the Tongrentang Drug Store (Beijing, China): *Astragalus mongholicus* Bunge [Fabaceae] (Huang Qi), *Citrus* × *aurantium* L. [Rutaceae] (Zhi Qiao), *Crataegus pinnatifida* Bunge [Rosaceae] (Shan Zha), *Schisandra chinensis* (Turcz.) Baill [Schisandraceae] (Wu Wei Zi), *Dendrobium officinale* Kimura & Migo [Orchidaceae] (Shi Hu), and *Angelica sinensis* (Oliv.) Diels [Apiaceae] (Dang Gui) with a dispensing proportion of 9:9:6:5:4:3. The ingredients were combined and boiled in distilled water for 30 min and then filtered. The solution was freeze-dried, ground into powder, and stored in a refrigerator at 4°C. Thereafter, the powder was accurately weighed and dissolved in 1 ml of 50% methanol water. Lysis was performed by sonication for 40 min at 23 ± 1°C. The solution was then centrifuged at 12,000 rpm for 10 min, and the supernatant was filtered through a 0.22-μm microporous membrane. Finally, the filtrate was injected into a liquid vial as a solution for ultra-high performance liquid chromatography-mass spectrometry (UHPLC-MS) testing, and the main KPLD compounds were identified.

Synapt G2-Si Qtof MS (Waters, Milford, MA, United States) and ACQUITY I Class-HPLC (Waters) were used to analyze the components of the KPLD extracts. In all chromatographic separations, an HSS T3 column (100 mm × 2.1 mm × 1.8 μm; Waters) was used at 35°C. The mobile phase was comprised of 0.1% formic acid solution (A) and 0.1% formic acid solution with acetonitrile (B) and had a flow rate of 0.25 ml/min. The gradient elution was as follows: 0–15 min, 0%–20% B; 15–50 min, 20%–100% B; 50–60 min, 100% B; 60–70 min, 100%–0% B. The MS utilized a 3 keV positive ionization voltage and 2.5 keV negative ionization voltage for electrospray ionization. The MS conditions were as follows: A sheath gas flow rate of 40 (arbitrary units), an auxiliary gas flow rate of 10 (arbitrary units), and a sweep gas flow rate of 3 μl/min were used, and a mass scanning range of 100–1,500 was employed. Compositional identification of each compound was compared to a theoretical database containing 6,400 natural products in the MS compound library.

### 2.2 Animals and treatment

Sixty SPF male Wistar rats (weight, 210 ± 10 g) were used in this study, all purchased from SPF Biotechnology Co., Ltd (Beijing, China), License number: SCXK 2019–0010. The animal feeding and experimental procedures were approved by the Animal Care and Use Committee of the Beijing University of Chinese Medicine (BUCM-4-2020120102-4093). The rats were left for five days to adapt to the environment (23 ± 1°C, 30%–40% relative humidity) with free access to water and food.

They were randomly divided into six groups of 10: control group, model group, CoQ10 group [Coenzyme Q10 (Maguire et al., 2020) of 10 mg/kg/d, positive drug], low dose group (LDG, KPLD crude of 3.24 g/kg/d), normal dose group (DG, KPLD crude of 6.48 g/kg/d), and high dose group (HDG, KPLD crude of 12.96 g/kg/d). Each herb of KPLD were obtained separately from the Tongrentang Drug Store (Beijing, China) and decocted in the lab. The administration dosage of the KPLD crude drug was calculated using the rat-to-human dosage relationship (Wei et al., 2010) based on the following conversion formula: crude drug dosage per kilogram of rats = adult drug dosage (g)/adult body weight × 6.3. The dose of KPLD extract was calculated as 510 mg/kg/d of DG, 255 mg/kg/d of LDG and 1.02 g/kg/d of HDG by calculating the human equivalent dose. The dosage of the DG was calculated from the clinical application dosage. The dosage of the LDG was half of that of the DG and that of the HDG was twice that of the DG. Rats in the control and CF model groups received the same amount of drinking water. Coenzyme Q10 was purchased from the China-Japan Friendship Hospital (Beijing, China). Drug administration was initiated on the 15th day of modeling. During the experiment, all animals that died due to model establishment, behavioral testing, anesthesia, or any other reason were not included as experimental observation subjects.

### 2.3 Establishment of the central fatigue model

**FIGURE 1:**
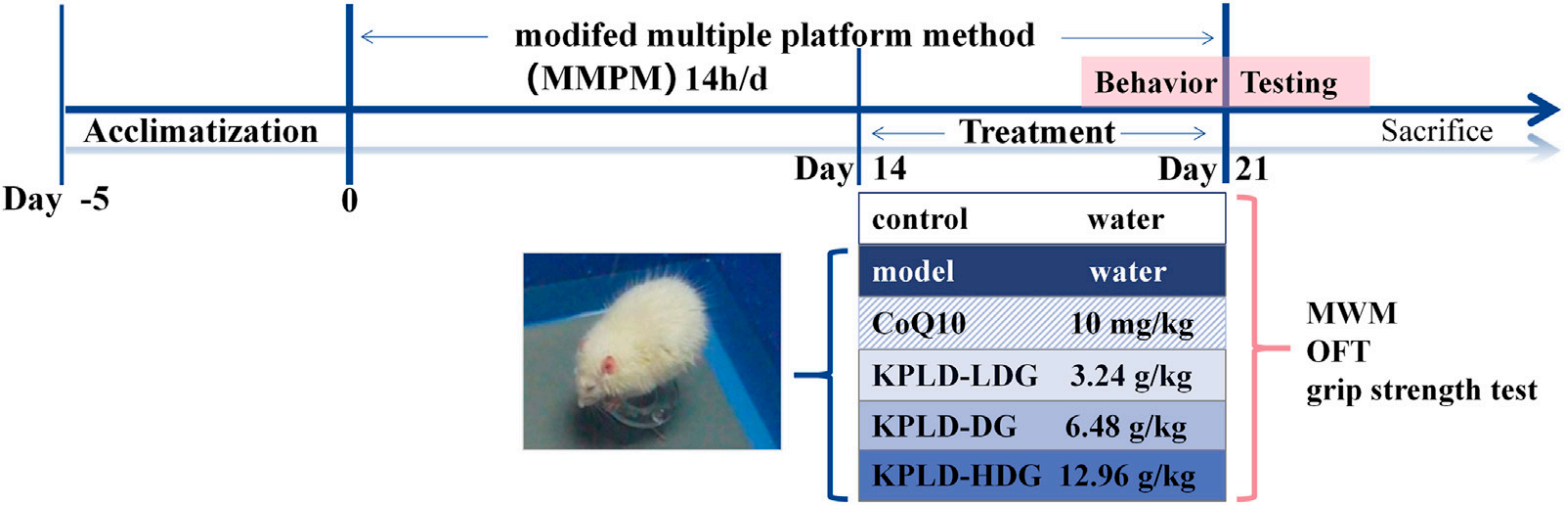
Experimental procedure and drug administration method.

To induce CF, we used the modified multiple platform method (MMPM) in all the rats except the controls (Zhang et al., 2018). We placed 15 platforms at the bottom of each plastic tank (110 × 60 × 40 cm) and filled the tanks with water at 22–25°C to 1 cm below the platform surfaces (Han et al., 2017). We left each rat in its own tank from 18:00 to 08:00 the next morning with access to food and water. After MMPM, rats were able to freely sleep, eat, and drink water until the next day before modeling. This procedure was repeated for 21 consecutive days. Figure 1 shows the experimental procedure and drug administration method.

### 2.4 Behavioral testing

The behavioral tests were all conducted the day following the training. After arriving in the testing room, each rat was allowed to acclimate for 1 h before testing commenced. The behavioral tests were performed as follows: grip strength, open-field test (OFT), and Morris water maze (MWM) with 2 h breaks between tests to minimize any adverse effects. All animals were tested at the same time. The video tracking system (SONY Inc., Japan) was placed above the apparatus to record and analyze the animals’ behavior using EthoVision XT (Noldus, Holland), a system used for analyzing the behavioral trajectory.

#### 2.4.1 Morris water maze

The MWM is a test of spatial learning and long-term memory for rats that utilizes distal cues to locate a submerged escape platform around the perimeter of an open swimming arena (Vorhees and Williams, 2006; D’Hooge and De Deyn, 2001). A circular tank (150 cm in diameter and 50 cm in depth) with a removable hidden platform (9 cm in diameter) submerged about 2 cm below the surface of the water (24 ± 1°C) served as the apparatus (Li et al., 2018). To provide location cues, different marker colors and shapes were placed, and each rat was trained four times for four consecutive days. During each training session, the rat was manually guided to the platform if the escape latency exceeded 60 s. On day 5 of the experiment, the platform was removed, and the rats were allowed to swim freely for 120 s.

#### 2.4.2 Open field test

An open-field experiment is a method used to evaluate the exploratory behavior, anxiety, and depression of experimental animals in a new environment (Prut and Belzung, 2003). The open-field arena (100 × 100 × 40 cm) was constructed with gray walls and a black floor and was divided into 25 equally sized squares, including the central and surrounding areas (Han et al., 2018b). Each rat was placed in the area for 5 min, during which we recorded the trajectory and measured the time in the central square, central percentage, total distance traveled, vertical activity, grooming behavior, and number of crossings. After each test, the equipment was wiped with a 75% alcohol solution.

#### 2.4.3 Grip strength

Each rat’s grip strength was quantitatively monitored using a grip strength meter (Biosep, France). The rats were placed over the instrument grid such that their claws fully grasped the grid. Their tails were pulled back horizontally and stably along the sensor axis until they released the grid. The apparatus was used in peak mode, and the recorded values corresponded to the maximum force generated by the animal. Grip strength was measured thrice, and the average value was recorded.

### 2.5 Blood serum analysis

The rats were euthanized using a deep intraperitoneal anesthetic injection of 10% pentobarbital sodium (4 ml/kg), and the blood was withdrawn from the abdominal aorta. The procedure strictly adhered to ethical requirements. Serum isolation was performed by centrifuging the blood at 3,000 rpm for 20 min at 4°C after collecting the blood in a blood collection tube. Serum levels of aspartate transaminase (AST), blood urea nitrogen (BUN), lactic acid (LAC), and lactate dehydrogenase (LDH) were measured with an automatic biochemical analyzer (Beckman Coulter, Brea, CA, United states) using standard laboratory methods. Glu and GABA serum concentrations were measured using Glu (CES122Ge; Cloud-clone Corp., Wuhan, China) and GABA (CEA900Ge; Cloud-clone Corp.) ELISA kits.

### 2.6 Hematoxylin and eosin staining

We fixed the prefrontal cortex and hippocampal tissues in 4% paraformaldehyde for 1 day, dehydrated them in a graded series of alcohols, subsequently embedded them in paraffin, and cut them into 5 mm-thick sections. The tissues were then stained with Hematoxylin and Eosin (H&E) according to routine protocols. Briefly, after dewaxing and rehydration, tissue sections were stained with hematoxylin solution (Servicebio, China) and rinsed. They were then stained with eosin solution (Servicebio, China), dehydrated with graded alcohol, and removed in xylene. Sections were assessed using an upright optical microscope (Nikon Eclipse E100, Tokyo, Japan).

### 2.7 Immunohistochemistry staining

The IHC staining distribution pattern of GABABR (Sheilabi et al., 2018) proteins was performed as previously reported. Brain sections were dewaxed with xylene and rehydrated using a reduced concentration of alcohol. The sections were placed in a repair cassette filled with citrate antigen repair buffer (pH 6.0) for microwave antigen repair then washed for 15 min and incubated with 0.3% H_2_O_2_ in phosphate buffer saline (PBS) for 25 min. Subsequently, the sections were rinsed three times with PBS (pH 7.4) for 5 min each, and the tissue was covered uniformly with 3% bovine serum albumin (BSA) dropwise for 30 min. GABABR2 (ab75838, 1:50; Abcam, Cambridge, United Kingdom) and NR2B antibodies (21920-1-AP, 1:100; Proteintech, Rosemont, IL, United states) were diluted in TRIS buffered saline solution (TBS) containing 1% BSA overnight at 4°C. Afterward, sections were washed in PBS and incubated with secondary antibodies for 50 min. After being washed thrice in PBS, the slices were incubated for 5–15 min with 0.05% diaminobenzidine tetrahydrochloride (DAB) solution in 50-mM Tris buffer (TB) containing 0.01% H_2_O_2_ at 22–25 °C for 10–12 min with controlled color development time under a microscope (Peyvandi et al., 2021). The slices were then counterstained for 3 min with hematoxylin, dehydrated with ethanol and xylene, and sealed.

### 2.8 Western blotting

The hippocampal and prefrontal cortex tissues were mixed with 10 times the volume of RIPA buffer, protease, and phosphatase inhibitor (KeyGEN, China) to extract brain tissue protein, and the protein concentration was measured by the bicinchoninic acid (BCA) method. Then, 50 μg of protein from each sample was loaded onto an SDS-PAGE gel of 10%–12% and electroblotted onto polyvinylidene fluoride (PVDF) membranes. After blocking the PVDF membranes with 4% skim milk in Tris-Buffered Saline and Tween (TBST) buffer for 1 h (Zhao et al., 2021), the following antibodies were incubated overnight at 4°C with the membranes: NMDAR2B (1:2000; Proteintech), GABABR1 (1:1,000; Abcam), GAT-1 (1:500; Abcam), GAD67 (1:1,000; Proteintech), GABAARα1 (1:2,000; Proteintech), GAGAARγ2 (1:1,000; Proteintech), and glyceraldehyde-3-phosphate dehydrogenase (GAPDH) (1:10,000, Proteintech). The membranes were then incubated with secondary antibodies (goat anti-rabbit IgG or goat anti-mouse IgG) for 2 h and washed thrice. A multifunctional molecular imaging system (Azure Biosystems, Dublin, CA, United states) was used for scanning and photography, and the Image J software was used for band gray analysis. In this study, all experiments were repeated three times with reproducible results.

### 2.9 Real-time quantitative polymerase chain reaction testing

Total RNA was extracted from the prefrontal cortex and hippocampal tissue using the Hipure Total RNA Mini Kit^®^ (MAGEN, China). The quality and quantity of RNA were measured using an Evolution201 spectrophotometer (Thermo Fisher Scientific, Waltham, MA, United states). The sequences of the primers used in RT-qPCR testing are listed in Table 1. RNA (2 μg) was converted to complementary DNA using the Reveraid First Strand cDNA Synthesis Kit (Thermo Fisher Scientific). RT-qPCR testing was performed using SYBR Green Master Mix on a Quantstudio^®^5 Real-time PCR instrument (Thermo Fisher Scientific). To normalize the variance between the samples, GAPDH was chosen as the endogenous control. Fold changes were calculated as 2−Δ(ΔCt) [ΔCt = Ct (target gene)—Ct (GAPDH); Δ(ΔCt) = ΔCt (experimental groups) − mean ΔCt (control groups)] (Lin et al., 2016).

**TABLE 1:** The primers for real-time RT-PCR. The primers of GABABR1, GABAARα1, GABAARγ2, GAD67, GAT-1, NR2B, GAPDH (as control) were presented in the table.

Genes	Forward	Reverse
GABABR1	5′-AGA​TTG​TGG​ACC​CCT​TGC​AC-3′	5′-AGA​AAA​TGC​CAA​GCC​ACG​TA-3′
GABAARα1	5′-AGT​GCG​ACC​ATA​GAA​CCG​AAA​G-3′	5′-TCC​AAA​TAG​CAG​CGG​AAA​GG-3′
GABAARγ2	5′-CCA​AAT​GAA​CAA​TGC​CAC​CCA​C-3′	5′-AAC​AAG​ATT​GAA​CAA​GCA​G-3′
GAD67	5′-GCG​GGA​GCG​GAT​CCT​AAT​A-3′	5′-TGG​TGC​ATC​CAT​GGG​CTA​C-3′
GAT-1	5′-TTC​CTG​ACG​CTC​ATC​TTT​GC-3′	5′-GAC​CAC​CTT​TCC​AGT​CCA​TC-3′
NMDAR2B	5′-CCT​GGA​ATG​GCA​TGA​TCG-3′	5′-AGC​CAC​CGC​AGA​AAC​AAT-3′
GAPDH	5′-GAC​ATG​CCG​CCT​GGA​GAA​AC-3′	5′-AGC​CCA​GGA​TGC​CCT​TTA​GT-3′

### 2.10 Statistical analysis

All the data were analyzed using SPSS 20.0 software and expressed as the mean ± standard deviation (SD). If the normal distribution was satisfied and the variance was homogeneous, the data was calculated and analyzed by one-way analysis of variance (ANOVA) and least significant difference; on the contrary, the Kruskal–Wallis test was used. Statistical significance was set at *p* < 0.05. Graphs were generated using GraphPad Prism eight (GraphPad, San Diego, CA, United States).

## 3 Results

### 3.1 Qualitative analysis of bioactive compounds in KangPiLao decoction

**FIGURE 2:**
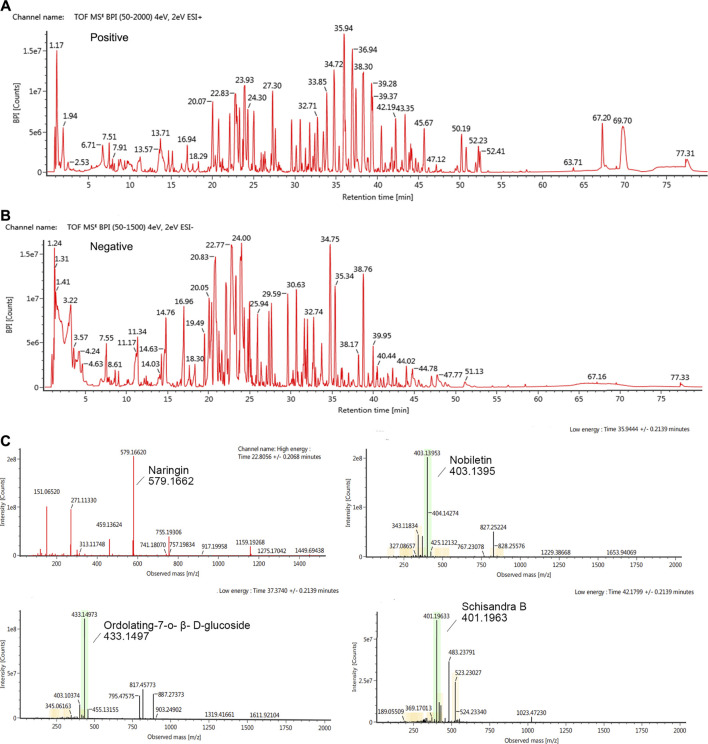
Ion chromatography of Bioactive Compounds in KPLD. Total ion chromatogram monitored in positive **(A)** and negative **(B)** ion modes for KPLD; KPLD composition analysis: KPLD has naringin, nobiletin, ordolating-7-o-β-D-glucoside, schisandra B and other ingredients **(C)**.

HPLC was used to determine the contents of the representative chemical components of KPLD. Figure 2 shows the total ion chromatography (TIC) on the positive mode (Figure 2A) and negative mode (Figure 2B) of KPLD. Compounds were qualitatively identified by comparing chromatography retention times and MS/MS data (Figure 2C). Finally, 36 main compounds were identified from KPLD, including naringin, nobiletin, Ordolating-7-o-β-D-glucoside, Schisandra B, and Dendrobium (Table 2).

**TABLE 2:** Analysis of the main chemical constituents of KPLD by UHPLC-MS/MS in positive and negative ion modes.

Identification	Formula	Calculated value	Observed value m/z	Error (ppm)	RT (min)	Peak area	Ion
Naringin	C_27_H_32_O_14_	580.17921	579.1662	−9.9	22.81	10,510,839	−H
Nobiletin	C21H22O8	402.13147	403.1395	1.9	35.94	6,617,944	+H
Naringenin	C15H12O5	272.06847	273.0767	3.5	22.8	4,006,854	+H
Ordolating-7-o-β-D-glucoside	C22H24O9	432.14203	433.1497	1	37.37	3,792,067	+H
Calycosin	C16H12O5	284.06847	285.0768	3.6	20.06	2,337,116	+H
Schisandra B (γ-Schisandrin)	C23H28O6	400.18859	401.1963	1.2	42.18	2,013,509	+H
Anacardin	C27H30O14	578.16356	579.171	0.3	22.89	2,006,726	+H
Schisandrin A	C24H32O6	416.21989	417.2278	1.5	50.19	2,000,546	+H
Formononetin	C16H12O4	268.07356	269.0818	3.7	32.74	1,779,579	+H
Hesperidin	C28H34O15	610.18977	611.1968	−0.4	23.92	1,656,361	+H
Luteolin-7-O-β-D-Neohesperidin	C27H30O15	594.15847	595.1661	0.7	16.93	1,645,112	+H
5-Hydroxylimidin	C20H20O8	388.11582	389.1239	2.1	40.45	1,547,274	+H
Dendrobium	C15H16O4	260.10486	261.1133	4.4	25.05	1,481,892	+H
Crotonyl Gomisin P	C28H34O9	514.22028	515.2282	1.3	37	1,192,735	+H
Isoliquiritin	C21H22O9	418.12638	419.1344	1.8	22.81	1,171,424	+H
Adenosine	C10H13N5O4	267.09675	268.1052	4.3	7.48	1,114,902	+H
5-Ortho-Demethyl Tetraritin	C20H20O8	388.11582	389.1237	1.7	32.4	1,031,560	+H
Neohesperidin	C28H34O15	610.18977	611.1963	−1.3	23.3	957,588	+H
Benzoyl gomisin H	C30H34O8	522.22537	523.2303	−4.5	42.18	903,290	+H
Aromaticin B	C17H20O6	320.12599	321.1336	1.1	1.2	852,493	+H
Adenine	C5H5N5	135.0545	136.0624	4.7	7.49	729,293	+H
Gomisin E	C28H34O9	514.22028	515.2279	0.6	35.34	661,844	+H
(−)-Gomisin K1	C23H30O6	402.20424	403.2123	2.1	44.64	536,213	+H
Gomisin J	C22H28O6	388.18859	389.1966	2	38.85	510,470	+H
Gomisin G	C30H32O9	536.20463	537.2097	−4.1	45.67	459,576	+H
Bergamot lactone	C12H8O4	216.04226	217.0503	3.6	32.19	435,691	+H
Gomisin F	C28H34O9	514.22028	515.2279	0.7	40.91	369,492	+H
Schisandrin A	C24H32O7	432.2148	433.2222	0.4	36.93	337,193	+H
Quercetin	C15H10O7	302.04265	303.0511	3.9	20.74	304,959	+H
Kaempferol	C15H10O6	286.04774	287.056	3.6	21.26	294,732	+H
E-Ligustilide	C12H14O2	190.09938	191.1073	3.5	41.25	131348	+H
Z-Ligustilide	C12H14O2	190.09938	191.1074	3.7	50.88	108,024	+H
Vanilla acetone	C9H10O3	166.06299	167.071	4.6	34.07	105,218	+H
Pinoresinol	C20H22O6	358.14164	357.1365	6.1	31.79	95,474	-H
Astragalus saponin I	C45H72O16	868.48204	869.4894	0.1	38.76	83,748	+H
Dendrobin C	C16H18O5	290.11542	289.1061	−7.1	7.2	70,580	−H

### 3.2 Effects of KangPiLao decoction on cognitive function of learning and memory

No rats died during modeling, but some of them developed tail, paw, and eye hemorrhages caused by irritability and aggression. These rats were excluded from the behavioral experiments. In addition, individual rats that showed cooperation difficulties, such as floating on the water surface and abnormal swimming patterns, were excluded from the MWM test.

**FIGURE 3:**
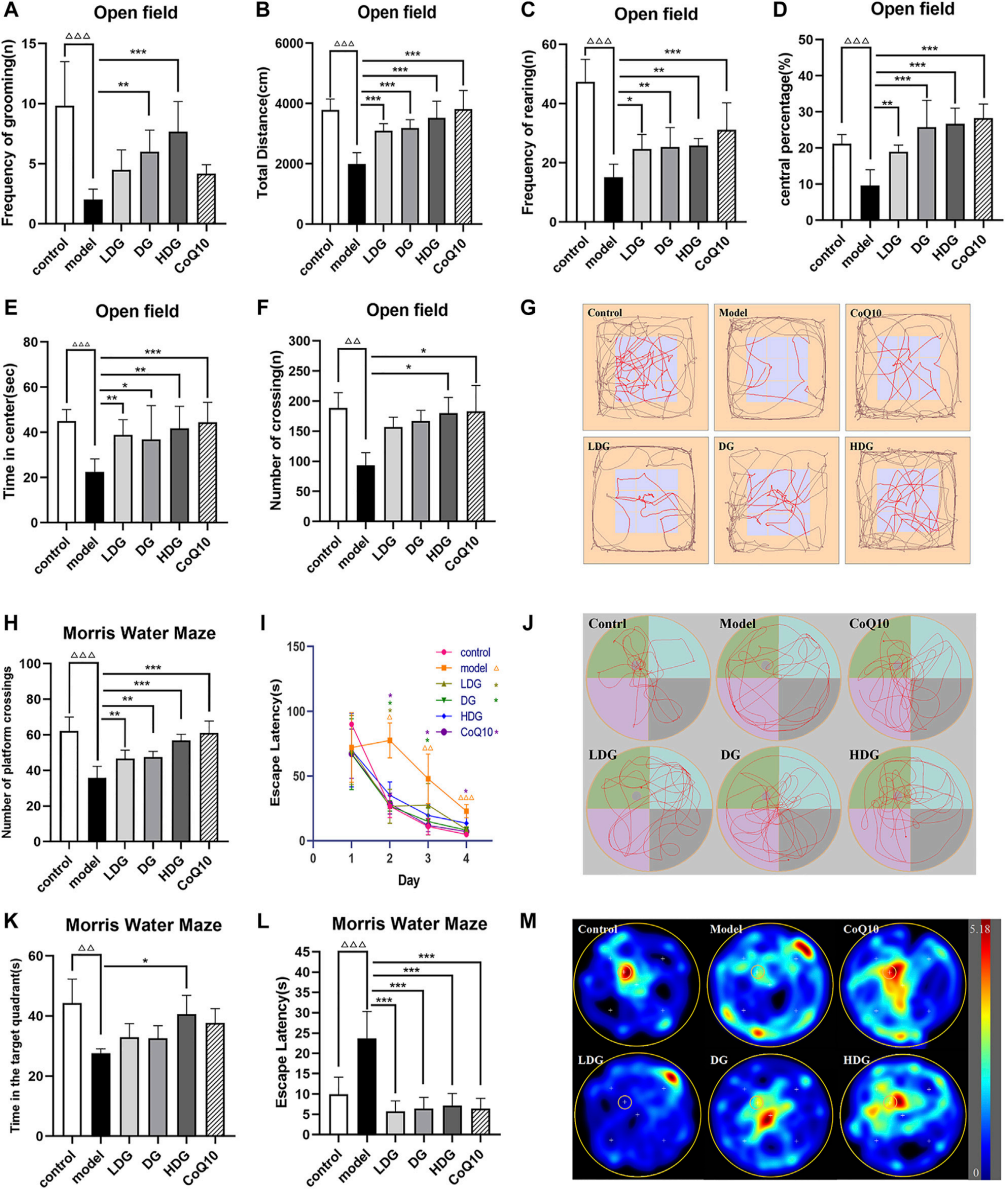
Effects of KPLD on emotion, spontaneous activity, and cognitive function of learning and memory evaluated by OFT and MWM tests. The total moving distance, time in the center, central percentage, number of crossing, frequency of rearing and grooming of rats on OFT **(A–F)**, and the map of representative movement trajectories in each group for 5 min in the open field **(G)** are shown. The numbers of platform crossings **(H)** and the time in target quadrant **(J)** on the last day during MWM test. The escape latencies in the first four consecutive days of positioning navigation training **(I)** and in the last day of spatial probe testing of finding hidden platforms **(K)** are shown. Representative swim paths during the spatial probe test **(L)** and heat map according to frequency and position of swimming paths **(M)**. Data were expressed as (mean ± SD), ^ΔΔ^ *p* < 0.01, ^ΔΔΔ^ *p* < 0.001 vs. control group; *p < 0.05, **p < 0.01, ***p < 0.001 vs. CF model group (*n* = 6).

In the MWM, the long-term memory and spatial learning of the rats were measured by the time spent in the target quadrant and escape latencies (Morris, 1984). In the trial, CF model rats had delayed escape latencies and spent less time in the target quadrant (*p* < 0.01 or *p* < 0.001, Figures 3I–K), indicating impaired cognitive function. The model rats also displayed a reduced number of platform passes (*p* < 0.001, Figure 3H). The therapeutic benefits were also observed in the LDG and DG (*p* <0.05, Figure 3I) as indicated by the escape latency observed in the first 4 days. When compared to the model group, the HDG showed increased swimming time in the target quadrant (*p* < 0.05). From the escape latency and number of crossings, each dose of KPLD and CoQ10 had obvious therapeutic effects on rats with CF (*p* < 0.01, Figures 3I–K). Among them, the HDG and CoQ10 groups showed significantly higher efficacy in improving the number of platform crossings (*p* < 0.001, Figure 3H). Figure 3L–M shows a representative trajectory of rats passing through different quadrants after removing the platform in the MWM test and a heat map of each group after superposition according to frequency and position. It can be seen from the spatial exploration trajectories that, except for the CF model group, the rats in the other groups adopted the trend-based search strategy learned after training. This evidence suggested that rats with CF displayed impaired spatial cognition and memory and that KPLD could significantly improve the cognitive function of rats.

### 3.3 Negative emotion and spontaneous activity in rats with central fatigue

The OFT is widely accepted as a reliable test of negative emotion-related motor activity in rodents (Jalewa et al., 2014). The results of the open field experiment showed that the total distance, time in the center, central percentage, frequency of rearing and grooming of the CF model of rats were significantly reduced (*p* < 0.001, Figures 3A–E). The number of crossings decreased significantly (*p* < 0.01, Figure 3F). According to the trajectory of the rats in the OFT, it was deduced that the CF model rats adopted marginal exploration. revealing that the HDG experienced better therapeutic benefits (Figure 3G). The HDG significantly improved all of the above indicators, and the HDG had a better effect than the other drug intervention groups in the improvement of modification times (Figure 3A). The OFT results suggested that rats with CF presented with decreased vitality and increased anxiety and depression. KPLD improved anxiety, depression, and spontaneous activity in rats with CF.

### 3.4 Peripheral fatigue in rats with central fatigue

**FIGURE 4:**
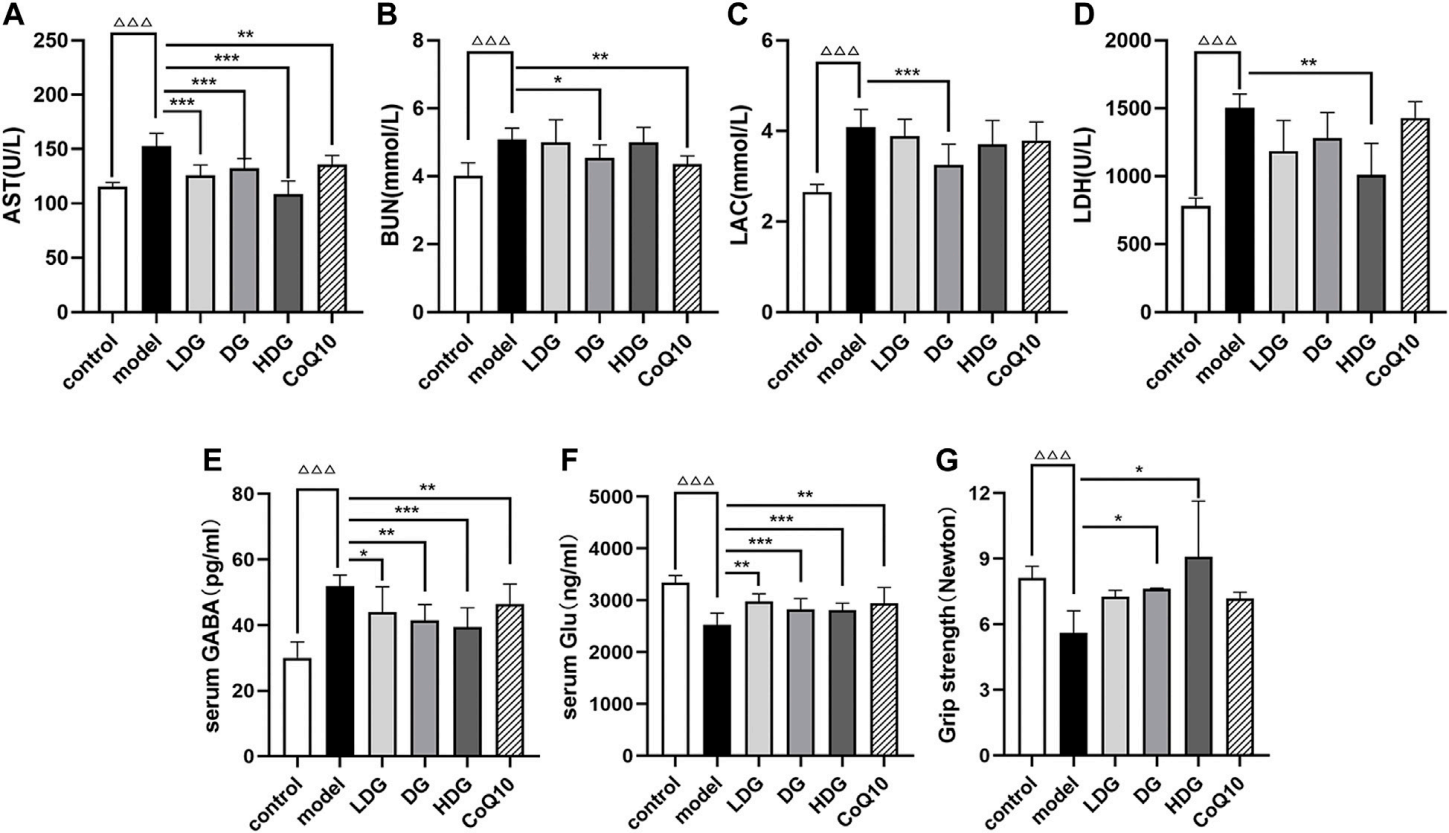
Effects of KPLD on peripheral fatigue and serum GABA and Glu levels in rats. Serum levels of AST **(A)**, BUN **(B)**, LAC **(C)** and LDH **(D)** were measured with an automatic biochemical analyzer. The expression levels of GABA **(E)** and Glu **(F)** in rat serum were tested by ELISA kits. Grip strength test was performed to detect skeletal muscle fatigue of rats **(G)**. Data were expressed as (mean ± SD), ^ΔΔΔ^ *p* < 0.001 vs. control group; *p < 0.05, **p < 0.01, ***p < 0.001 vs. CF model group (*n* = 7).

Figures 4A–D show that the serum AST, BUN, LAC, and LDH levels of rats with CF were significantly higher than control group (*p* < 0.001), suggesting a certain degree of physical fatigue and liver injury risk. Both the KPLD high-medium-low-dose and CoQ10 groups showed significantly reduced serum AST levels (*p* < 0.001 or *p* < 0.01, Figure 4A). DG and CoQ10 treatments effectively reduced the BUN content (*p* < 0.05, *p* < 0.01, Figure 4B). In addition, the DG and HDG showed significantly decreased serum LAC (*p* < 0.001, Figure 4C) and LDH levels (*p* < 0.01, Figure 4D, *n* = 7).

In addition, we performed a grip strength test to detect skeletal muscle fatigue in each rat group (Figure 4G). The experimental results showed that the grip strength value in the CF group decreased significantly (*p* < 0.001), suggesting that the CF model of rats experienced skeletal muscle fatigue and medium- to high-dose KPLD could improve skeletal muscle fatigue (*p* < 0.05; n = 6).

### 3.5 Morphology changes associated with KangPiLao decoction treatment

**FIGURE 5:**
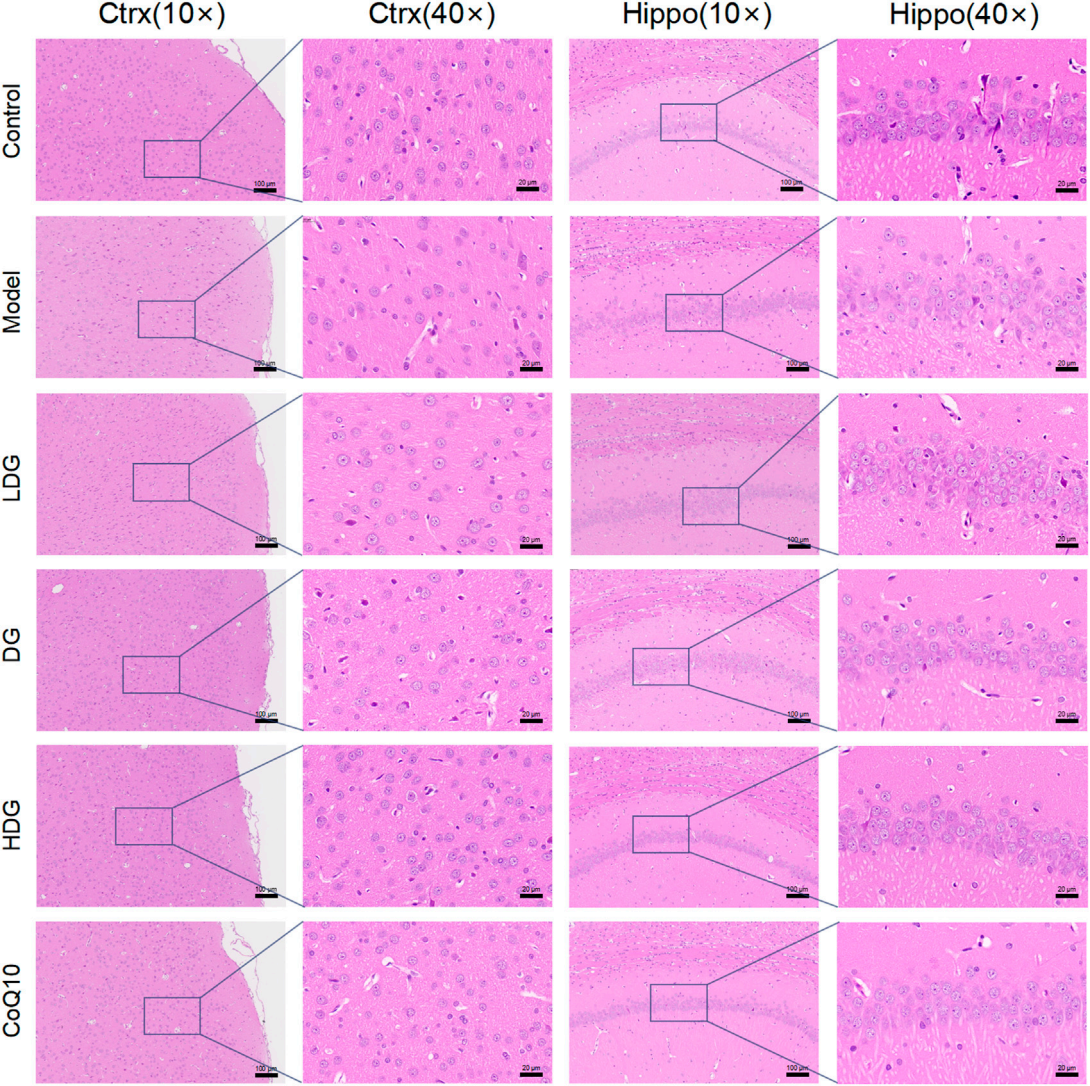
Histological changes associated with KPLD Treatment. H&E staining of the prefrontal cortex (**A**: ×10, ×40 magnification) and hippocampal CA1 region (**B**: ×10, ×40 magnification) of the rats were observed by light microscopy.

Figure 5 shows the effect on tissue morphology of the hippocampus and prefrontal cortex with KPLD treatment. H&E staining of the prefrontal cortex (Figure 5A) and hippocampal CA1 region (Figure 5B) of the rats was observed under a microscope. Nissl bodies are characteristic structures of neurons and thus were observed by light microscopy to identify neurons. Hippocampal CA1 glial cells in the control group were tightly arranged and normal in shape. The formation of new neurons was observed under the microscope. In contrast, glial cells in the model group were loosely arranged, cells were missing, some nuclei were condensed and deeply stained, and the structure was blurred. In addition, the number of pyramidal cells in the prefrontal cortex and hippocampus of the rats in the model group was reduced. The morphology of glial cells in the middle-high-dose treatment groups and the CoQ10 group improved, and the number of pyramidal cells and Nissl bodies increased to a certain extent with KPLD.

### 3.6 Regulation of serum gamma-aminobutyric acid and glutamic acid levels by KangPiLao decoction

We measured the expression levels of Glu and GABA in rat serum using ELISA kits (Cloud-Clone Corp., Wuhan, China). GABA levels were significantly increased in the model group (*p* < 0.001, Figure 4E). Treatment with the KPLD decoction and CoQ10 significantly decreased GABA levels, with the most significant improvement observed in the HDG (*p* < 0.001). Glu levels were significantly lower in rats with CF (*p* < 0.001, Figure 4F). Both KPLD and CoQ10 interventions increased Glu levels, with the DG and HDG showing the most significant improvement (*p* < 0.001; *n* = 7).

### 3.7 Regulation of the gamma-aminobutyric acid/glutamic acid pathway protein expression by KangPiLao decoction

**FIGURE 6:**
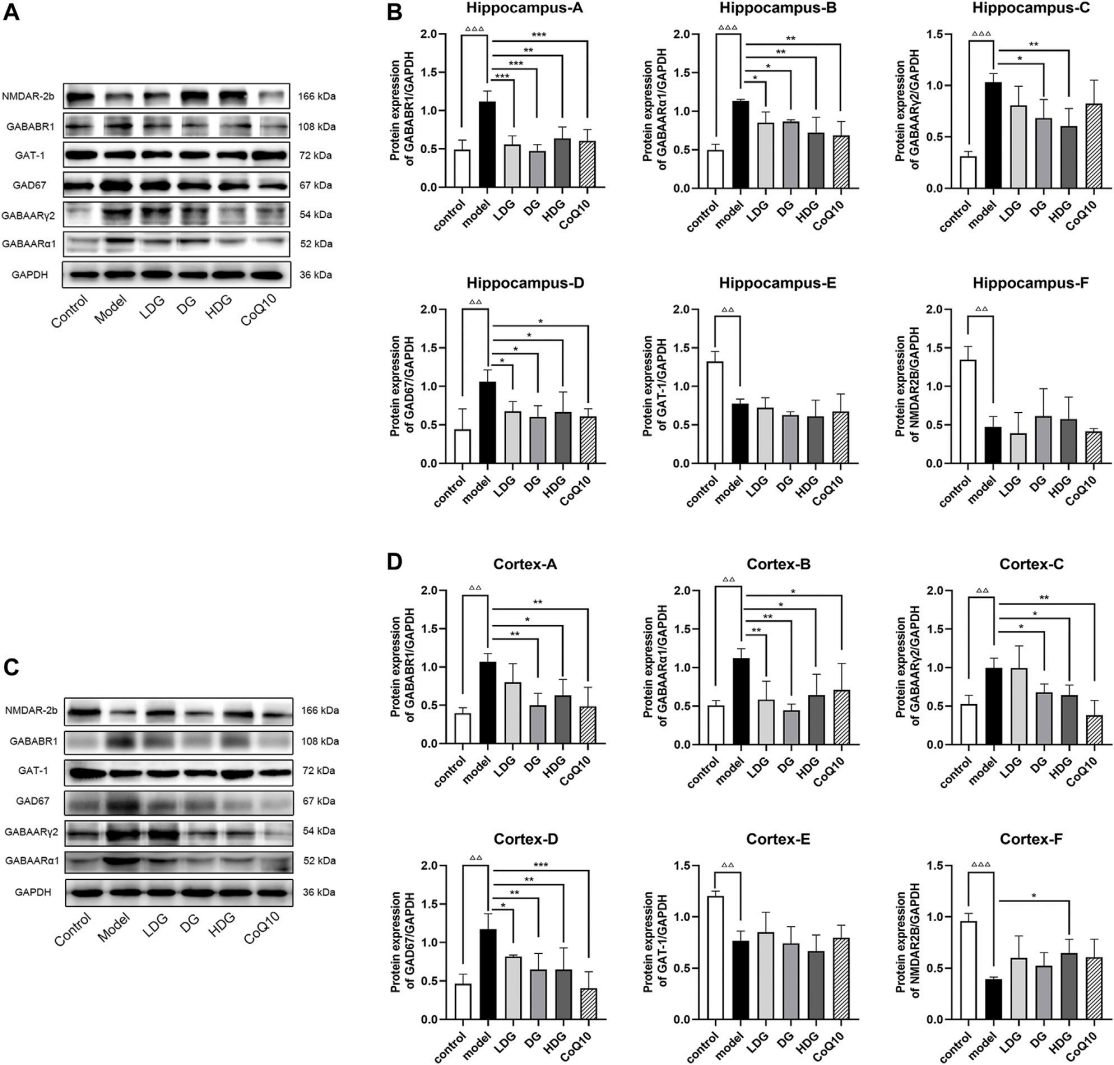
Regulation of GABA/Glu pathway-related protein expression levels in all groups. The representative images in hippocampus **(A)** and prefrontal cortex **(C)** and the protein levels of GABAARα1, GABAARγ2, GABABR1, GAD67, GAT-1 and NR2B in hippocampus **(B)** and prefrontal cortex **(D)** are shown. Data were expressed as (mean ± SD), ^ΔΔ^ *p* < 0.01, ^ΔΔΔ^ *p* < 0.001 vs. control group; *p < 0.05, **p < 0.01, ***p < 0.001 vs. CF model group (n = 3).

We detected the expression levels of GABA/Glu pathway-related proteins using western blotting (Figure 6). GABAARα1, GABAARγ2, GABABR1, and GAD67 protein expression levels were significantly increased (*p* < 0.001, *p* < 0.01), whereas GAT-1 and NR2B protein expression levels were significantly decreased in the hippocampus and prefrontal cortex regions of rats with CF (*p* < 0.01). Compared to the model group, both the KPLD medium-high-dose group and CoQ10 reduced the protein expression of GABAARα1, GABABR1, GAD67, and GABAARγ2 in the prefrontal cortex and hippocampus (*p* < 0.05). In addition, the HDG showed decreased GABAARγ2 protein expression in the hippocampus of rats with CF (*p* < 0.01) and increased NR2B protein expression levels in the prefrontal cortex (*p* < 0.05; *n* = 3).

**FIGURE 7:**
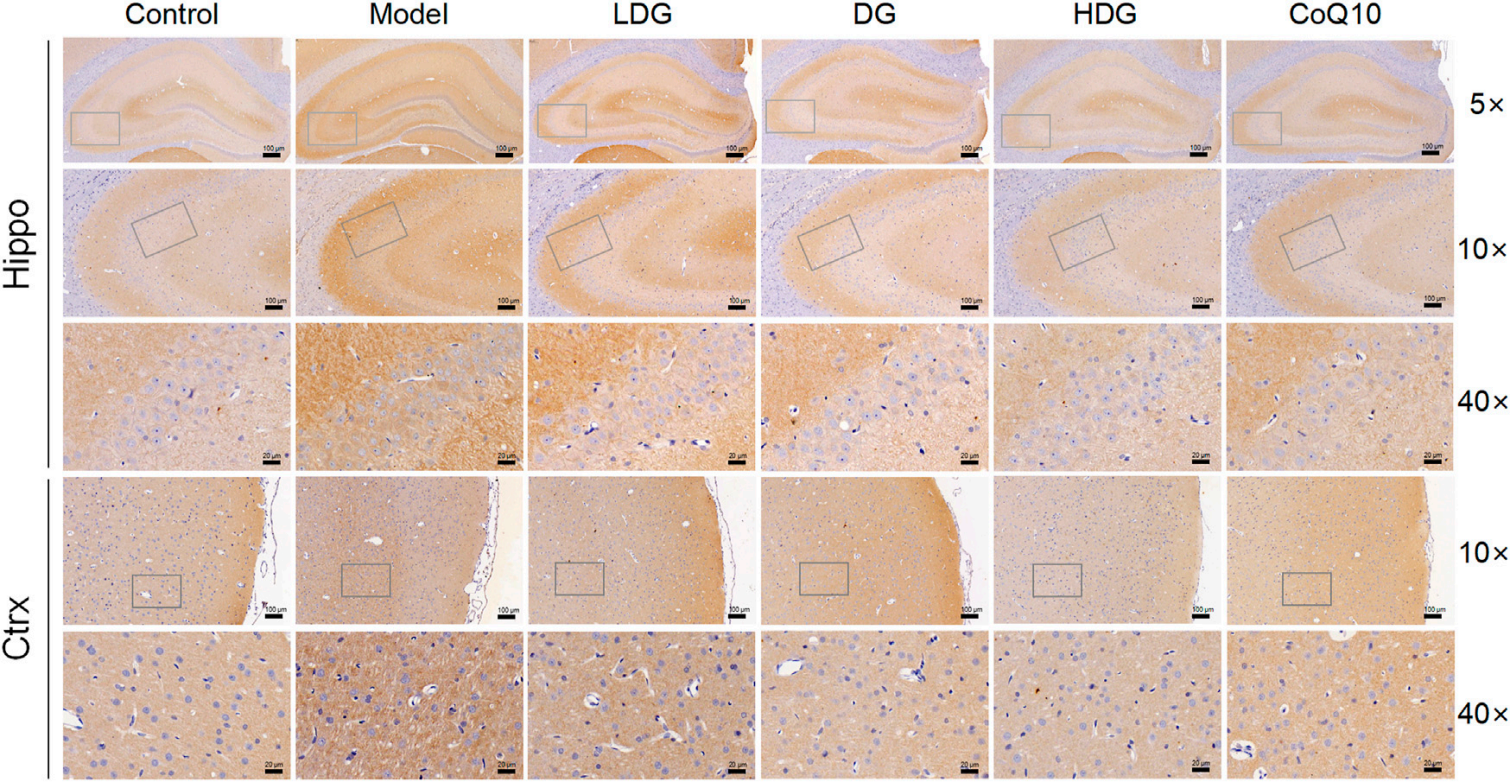
Immunohistochemical staining for GABABR2 in the hippocampal CA3 region (×5, ×10, ×40 magnification) and the prefrontal cortex stromal layer (×10, ×40 magnification) of rats.

**FIGURE 8:**
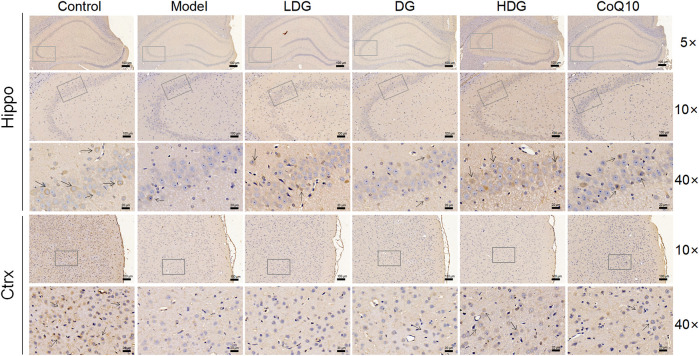
Immunohistochemical staining for NMDAR2B in the hippocampal CA3 region (×5, ×10, ×40 magnification) and the prefrontal cortex stromal layer (×10, ×40 magnification) of rats.

We detected GABABR2 and NMDAR2B (NR2B) expression in the hippocampus and prefrontal cortex by IHC. The staining of GABABR2 (Figure 7) in the glial cell envelope, stroma, and prefrontal stromal layer of the CA3 region of rats with CF deepened, with the expression significantly higher than that in the normal group. The KPLD and CoQ10 groups stained lighter than the model group, which could reduce the level of overexpressed GABABR2. In contrast, the number of positive cells and the expression of NR2B (Figure 8) in the hippocampal CA3 region and prefrontal glial cell envelope of rats with CF were lower, and the improvement of NR2B by KPLD was not obvious.

### 3.8 Gene expressions of gamma-aminobutyric acid/glutamic acid pathway in hippocampus and cortex

**FIGURE 9:**
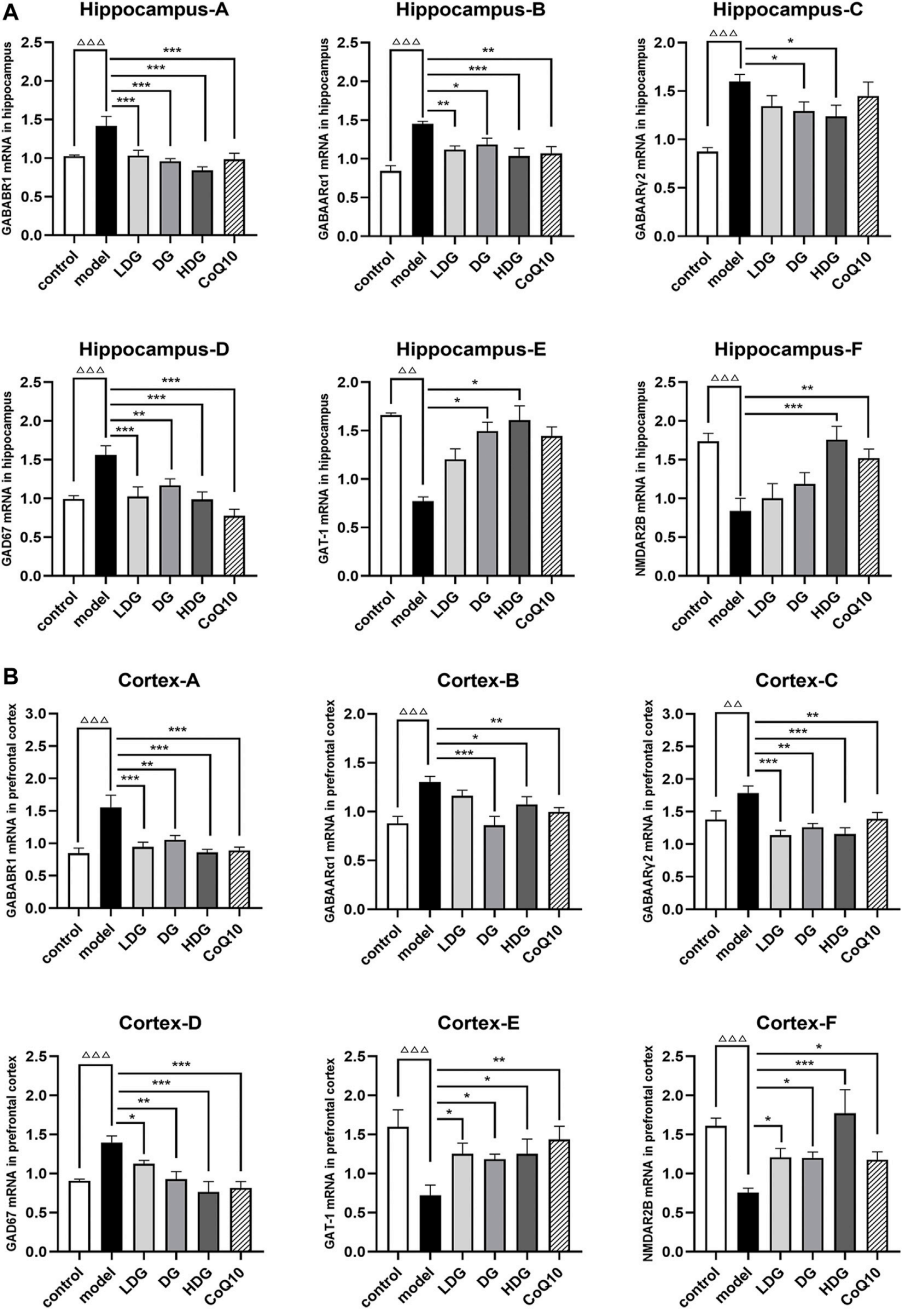
Expression levels of GABA/Glu pathway-related genes in the hippocampus and cortex. Relative mRNA expression levels of GABAARα1, GABAARγ2, GABABR1, GAD67, GAT-1 and NR2B in the hippocampus **(A)** and prefrontal cortex **(B)** were detected by real time-PCR analysis respectively. Data were expressed as (mean ± SD), ^ΔΔ^ *p* < 0.01, ^ΔΔΔ^ *p* < 0.001 vs. control group; *p < 0.05, **p < 0.01, ***p < 0.001 vs. CF model group (*n* = 6).

The relative mRNA levels of GABAARα1, GABAARγ2, GABABR1, GAD67, GAT-1, and NR2B in the hippocampus (Figure 9A) and prefrontal cortex (Figure 9B) were detected using RT-qPCR. We observed a significant upregulation of GABAARα1, GABAARγ2, GABABR1, and GAD67 and downregulation of GAT-1 and NR2B in rats with CF (*p* < 0.01, *p* < 0.001). In contrast, GABAARα1, GABAARγ2, GABABR1, and GAD67 were downregulated (*p* < 0.05), and GAT-1 and NR2B were upregulated (*p* < 0.05) in the KPLD and CoQ10 treatment groups. The high-dose KPLD group upregulated NR2B expression significantly in the hippocampus and prefrontal lobes of rats with CF (*p* < 0.001; *n* = 6).

## 4 Discussion

CF is induced by strenuous physical or mental tasks, leading to cognitive and memory impairment, reduced physical endurance, negative emotions (e.g., anxiety, depression, hostility), and various metabolic disturbances in the central and peripheral systems (Harrington, 2012). The current research hypotheses related to the abnormal regulation of the neuroendocrine system and neurotransmitters in the manifestation of cognitive fatigue are 1) Abnormal reward mechanisms involving dopamine. It has been found that the underlying motivational system that drives the brain to work is the subconscious reward system (Boksem and Tops, 2008), including liking, wanting, and learning and that fatigue occurs when this mechanism is abnormal (Hauber, 2010); 2) Hypothalamic-pituitary-adrenal (HPA) axis disorders. The HPA axis is the main sensing system of the body when stress is experienced. Long-term stress, insomnia, and other mental factors affect the neuroendocrine system to produce a series of changes that eventually leads to the onset of fatigue (Kempke et al., 2015); 3) Compensation effects. When cognitive work is overloaded and efficiency decreases, the CNS inhibits negative feedback regulation to compensate for decreased work efficiency, which leads to fatigue (Lewis and Moghaddam, 2006); and 4) Imbalance in the excitation/inhibition pathway regulation. When there is an imbalance in the inhibition-excitation pathway in the CNS, fatigue occurs (Leavitt and Deluca, 2010).

**FIGURE 10:**
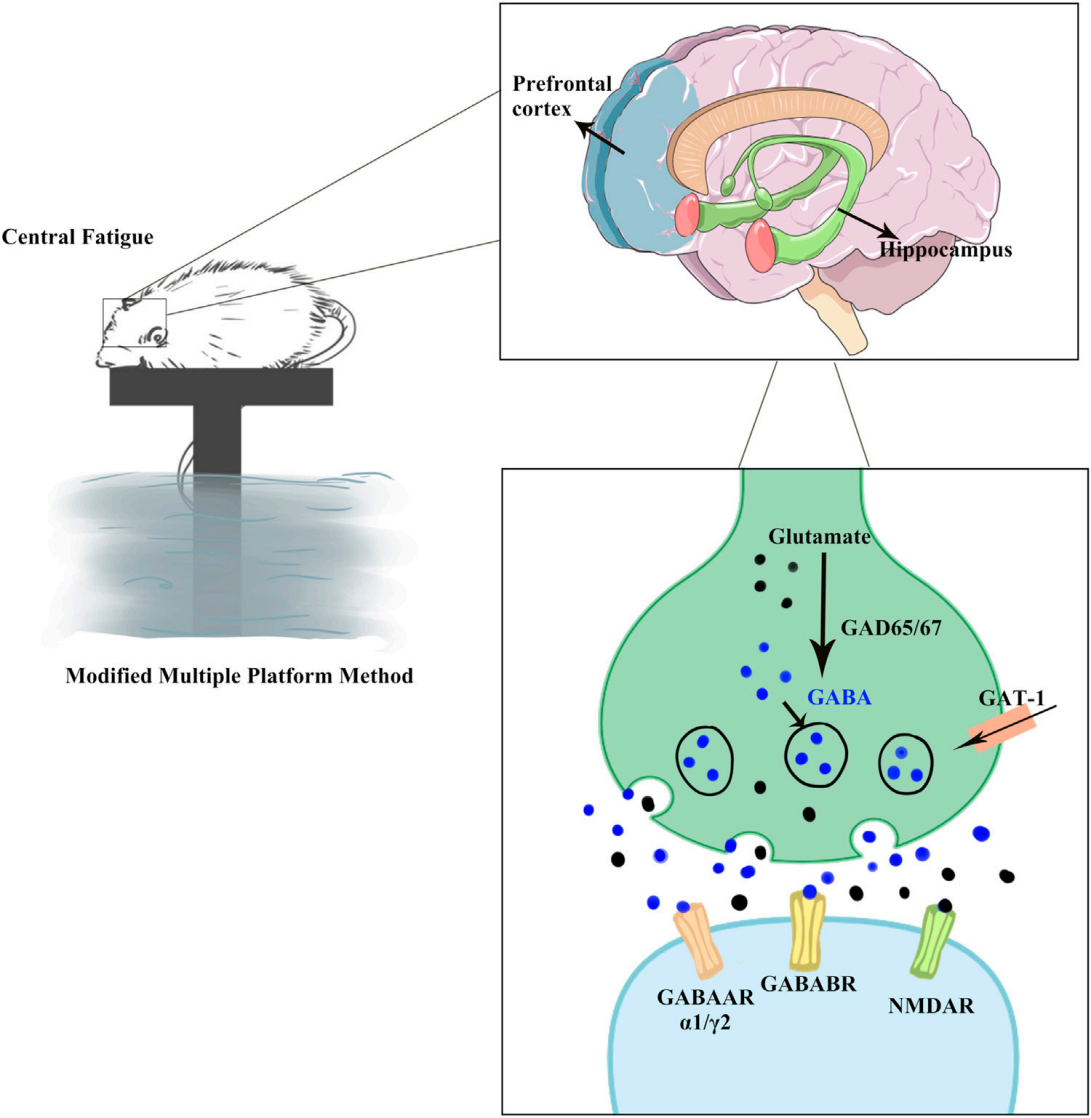
GABA/Glu signaling pathway prediction. CF over-activated the GABAergic system and inhibited the Glu-ergic system, resulting in hippocampal and prefrontal cortex damage, as well as cognitive and emotional disorders in rats. KPLD could be used to regulate the changes in Glu and GABA levels as well as improve the cognitive, emotional, and physical disorders of rats with CF. GABAARα1 and GABAARγ2, as subunits of GABAAR, are more widely distributed in the hippocampus and cortex. GABABR is a heterodimer comprising of GABABR1 and GABABR2 subunits. GABA is formed through Glu conversion, which is mediated by the rate-limiting enzyme GAD. GAD67, an important isozyme, is involved in the synthesis of GABA. GAT-1 is a GABA transporter that regulates GABA reuptake to reduce GABA levels. NMDAR2B, as receptor of Glu, is of significant importance in dynamic processes such as learning and memory.

Glu and GABA are considered the major excitatory and inhibitory neurotransmitters in the CNS, respectively (Figure 10). Studies have identified the homeostatic mechanisms associated with cognitive and emotional functions (Lewis and Moghaddam, 2006). GABA synthesis in the brain relies on the conversion of Glu (Kuriyama and Sze, 1971), an excitatory neurotransmitter and raw material for the production of GABA, which is produced by the action of the glutamate decarboxylase GAD. Under normal conditions, the secretion of Glu and GABA in the brain is balanced.

GABAAR is a heterodimeric structure with 19 different subunits (Rudolph et al., 2001), of which GABAARα1 and GABAARγ2 are more widely distributed in the hippocampus and cortex. Blocking GABAAR can lead to changes in neural responses, such as anxiety and altered voluntary activity (Shekhar and Katner, 1995; Asinof and Paine, 2013; Kangarlu-Haghighi et al., 2015). GABABR is a heterodimer comprising GABABR1 and GABABR2 subunits (Pin and Bettler, 2016). Studies have shown that GABABR is associated with learning memory (Getova et al., 1997; Fujita et al., 2011). As an important inhibitory signal, GABAB receptor-mediated disruption of the GABAergic system is associated with many neurological and neuropsychiatric disorders such as anxiety, depression, and epilepsy (Ramamoorthi and Lin, 2011). GABA is formed through Glu conversion, which is mediated by the rate-limiting enzyme GAD. The content of GAD can reflect GABA concentration to some extent, and GAD67, an important isozyme, is involved in the synthesis of GABA (Sloviter et al., 1996). GAT-1 is a GABA transporter that regulates GABA reuptake to reduce GABA levels (Jensen et al., 2003). Excitatory transmission in neurons may be regulated by the number and composition of Glu receptors. Functional NMDA receptors are mainly composed of NR1 and NR2 subunits in a certain ratio (Hardingham and Bading, 2010). Among them, NR2B is an important subunit involved in the formation of the NMDA receptor and plays a key role in NMDA receptor function. Many studies on neurodegenerative diseases and mental diseases have found that the NR2B subunit exhibited abnormal function (Wang et al., 2011). One such receptor, NMDAR2B, is of significant importance in dynamic processes such as learning and memory (Pérez-Otaño and Ehlers, 2004).

We found that the protein and mRNA expression of GABAARα1, GABAARγ2, GABABR1, and GAD67 were significantly higher, while GAT-1 and NR2B were lower in the hippocampus and prefrontal lobe of rats with CF. The change in GABA and Glu receptor levels in the serum of CF model rats is consistent with the changes of GABA and Glu receptor levels in the brain. IHC revealed increased staining of GABABR2 in the glial cell envelope, stroma, and prefrontal stromal tissue layer in the CA3 region of the hippocampus and decreased expression of NMDAR2B. In some studies related to cognitive fatigue, the expression of GABA/Glu transmitter receptors showed an opposite trend (Gao et al., 2018). Gao et al. found that the reduced verbal memory and visuospatial memory observed in patients with MS correlated with decreased levels of GABA and Glu, respectively, by combining J-difference-edited magnetic resonance spectroscopy and resting-state functional magnetic resonance imaging. However, the different results may be due to the differences between research methods and the different diseases being studied. CF may be the early stage of a series of cognitive memory disorders associated with the bidirectional regulation of GABA/Glu transmitters. In the later stage with further aggravation of neurons, glial cells, and other tissue damage, the compensatory ability could not be adjusted. KPLD reduced the protein and mRNA expression of GABAARα1, GABABR1, GAD67, and GABAARγ2 in the prefrontal cortex and hippocampus. KPLD also increased the protein and mRNA expression of NR2B in the prefrontal cortex and GAT-1 mRNA in the prefrontal cortex and hippocampus, which is advantageous compared with CoQ10. Related studies have also shown that the CNS of rats is inhibited after fatigue, with a significant increase in the GABA content (Blanco-Lezcano et al., 2017). In addition, the improvement of NR2B and GAT-1 by drugs is not obvious, and some studies have reported similar results (Sta et al., 2017), which may be related to drug activation sites and receptor sensitivity. Activation of the relevant receptors could be studied in greater depth in the future.

We also found an association between CF and glial cells where H&E staining showed a loosely disorganized arrangement of glial cells, cellular deficits, solidified and deeply stained nuclei, and blurred structures in hippocampal and prefrontal cortical organization. Rönnbäck and Hansson (2004) found that in a state of mental fatigue, there is swelling of astrocytes, which in turn reduces Glu transmission. Le Meur et al. (2012) found that hippocampal astrocytes released GABA under the dual influence of both GABAergic and glutamatergic systems. We found that KPLD ameliorated glial cell damage in the hippocampus and prefrontal cortex of rats with CF to a certain extent. In addition, studies have found that astrocytes and microglia act together in the CNS and play an important role in managing neuronal development and alleviating cognitive impairment (Vainchtein and Molofsky, 2020). The correlation between CF and glial cells should be studied further in the future.

It was further reported in this study that decreased cognitive function and increased negative affect are the main manifestations of CF, which can be reflected by behavioral indicators (Leavitt and Deluca, 2010). The MWM detects cognitive and memory abilities of rats using target quadrant swimming time, escape latency, number of platform crossings, and spatial exploration strategy indicators. Emotional and arousal states are reflected in the OFT, such as the total distance, time in the center, central percentage and frequency of rearing and grooming (Zhuang et al., 2014; Guo et al., 2015). When rats were in a state of depression and anxiety, their locomotor distance was significantly shortened, and the time spent in the center decreased (Winocur, 1985). We found that the MWM results in the CF group of rats showed impaired spatial learning, and memory and the indicators of the OFT exhibited a higher negative effect. KPLD treatment significantly improved negative emotional impact and cognitive dysfunction, especially in the high-dose group.

At the same time, physical fatigue is closely related to CF (Martin et al., 2010; Temesi et al., 2014), which leads to the slowing of oxidative processes and energy metabolite consumption, as well as reduced muscle strength and efficiency (Waersted and Westgaard, 1996; Bloemsaat et al., 2005). Serum BUN, LDH, LAC, and AST levels and grip strength reflected the degree of body fatigue. Among them, LAC and BUN reflected the degree of physical and CF (Sorichter et al., 1999; Draper et al., 2006; Han et al., 2018b), LDH indicated the state of muscle function (Coqueiro et al., 2018), and an elevated AST reflected exercise fatigue (Luo et al., 2019). We found that serum AST, BUN, LAC, and LDH levels in rats with CF increased and grip strength decreased, suggesting the existence of central and physical fatigue. Our experimental results showed that KPLD improved physical fatigue by reducing serum AST, BUN, LAC, and LDH levels, more prominently than CoQ10.

Our research confirmed that there is an imbalance between GABA and Glu regulation in CF. The transmission of the neuronal synaptic transmitter GABA is mediated by GABAARα1, GABAARγ2, and GABABR and exerts an inhibitory effect. In an abnormal state, cognitive function declined. Symptoms such as increased negative emotions were consistent with the behavioral performance of rats with CF in this experiment. For example, in our water maze test, the time taken by CF rats to find a platform increased, and the time taken to swim in the target quadrant decreased, indicating negative emotions. In contrast, Glu relied on NMDAR2B and other mediators to exert excitatory effects. This experiment found that KPLD improved behavioral indicators by regulating GABA/Glu-related receptor protein and gene expression more than with CoQ10. In addition, KPLD is composed of medicine and food homologous herbal ingredients that might slow down further cognitive impairment and be potentially used in the early prevention of central fatigue. Studies have confirmed that naringin (Yang et al., 2020), nobiletin (Wang et al., 2019), schisandra (Kim et al., 2017) and other major compounds detected in KPLD can inhibit neuronal apoptosis and inflammation, reduce neurotoxicity, and play a protective role in nerve injury. It is worth noting that although the experiment highlights the positive effects of the HDG on mood and cognitive function relative to the CoQ10 group, the dose administered in the HDG is higher than the conventional dose used clinically. Thus, further studies are required to determine the optimal clinical usage conditions of KPLD.

Nevertheless, there are still some limitations to this study. First, the observation period of the CF model study was short; therefore, the results of this experiment were limited to the model, which could be later optimized. Second, only the key indicators were selected in this experiment, and the scope of the research can be expanded in the future. Finally, the internal mechanism involved in this experiment was relatively shallow, and the correlation between glial cells and the GABA/Glu pathway should be studied further.

## 5 Conclusion

We noted that there was over-activation of the GABAergic system and inhibition of the Glu-ergic system in CF and that KPLD could regulate the changes in Glu and GABA levels, as well as improve the cognitive, emotional, and physical disorders of rats with CF. KPLD has advantages in the regulation of Glu-related transmitter receptors and in improving cognitive impairment and physical fatigue. The beneficial effects of KPLD may be mediated by the activation of the GABA/Glu signaling pathway.

## Data Availability

The original contributions presented in the study are included in the article/supplementary material, further inquiries can be directed to the corresponding author.

## Glossary

AST: aspartate transaminase
ANOVA: analysis of variance
BCA: bicinchoninic acid
BSA: bovine serum albumin
BUN: blood urea nitrogen
CF: central fatigue
CNS: central nervous system
CoQ10: Coenzyme Q10
DAB: diaminobenzidine tetrahydrochloride
DG: normal dose group
GABA: gamma-aminobutyric acid
GAPDH: glyceraldehyde-3-phosphate dehydrogenase
Glu: glutamic acid
H&E: Hematoxylin and Eosin
HDG: high dose group
HPA: Hypothalamic-pituitary-adrenal
HPLC-MS: high-performance liquid chromatography-mass spectrometry
IHC: immunohistochemistry
KPLD: KangPiLao decoction
LAC: lactic acid
LDG: low dose group
LDH: lactate dehydrogenase
MMPM: modified multiple platform method
MWM: Morris water maze
OFT: open field test
PBS: phosphate buffer saline
PVDF: polyvinylidene fluoride
SD: standard deviation
TB: Tris buffer
TBS: Tris-buffered saline solution
TBST: Tris-buffered saline and Tween
TIC: total ion chromatography
